# The Increase in Animal Mortality Risk following Exposure to Sparsely Ionizing Radiation Is Not Linear Quadratic with Dose

**DOI:** 10.1371/journal.pone.0140989

**Published:** 2015-12-09

**Authors:** Benjamin M. Haley, Tatjana Paunesku, David J. Grdina, Gayle E. Woloschak

**Affiliations:** 1 Department of Radiation Oncology, Northwestern University, Feinberg School of Medicine, Chicago, Illinois, United States of America; 2 Departments of Radiation and Cellular Oncology, University of Chicago, Chicago, Illinois, United States of America; 3 Departments of Radiation Oncology, Radiology, and Cell and Molecular Biology, Northwestern University, Feinberg School of Medicine, Chicago, Illinois, United States of America; University of Oklahoma Health Sciences Center, UNITED STATES

## Abstract

**Introduction:**

The US government regulates allowable radiation exposures relying, in large part, on the seventh report from the committee to estimate the Biological Effect of Ionizing Radiation (BEIR VII), which estimated that most contemporary exposures- protracted or low-dose, carry 1.5 fold less risk of carcinogenesis and mortality per Gy than acute exposures of atomic bomb survivors. This correction is known as the dose and dose rate effectiveness factor for the life span study of atomic bomb survivors (DDREF_LSS_). It was calculated by applying a linear-quadratic dose response model to data from Japanese atomic bomb survivors and a limited number of animal studies.

**Methods and Results:**

We argue that the linear-quadratic model does not provide appropriate support to estimate the risk of contemporary exposures. In this work, we re-estimated DDREF_LSS_ using 15 animal studies that were not included in BEIR VII’s original analysis. Acute exposure data led to a DDREF_LSS_ estimate from 0.9 to 3.0. By contrast, data that included both acute and protracted exposures led to a DDREF_LSS_ estimate from 4.8 to infinity. These two estimates are significantly different, violating the assumptions of the linear-quadratic model, which predicts that DDREF_LSS_ values calculated in either way should be the same.

**Conclusions:**

Therefore, we propose that future estimates of the risk of protracted exposures should be based on direct comparisons of data from acute and protracted exposures, rather than from extrapolations from a linear-quadratic model. The risk of low dose exposures may be extrapolated from these protracted estimates, though we encourage ongoing debate as to whether this is the most valid approach. We also encourage efforts to enlarge the datasets used to estimate the risk of protracted exposures by including both human and animal data, carcinogenesis outcomes, a wider range of exposures, and by making more radiobiology data publicly accessible. We believe that these steps will contribute to better estimates of the risks of contemporary radiation exposures.

## Introduction

### Why radiation matters

Ionizing radiation exposure is a ubiquitous health risk. The National Council on Radiation Protection (NCRP) estimates that Americans were exposed to 2.7 million Sieverts (Sv) of non-therapeutic ionizing radiation in 2006 alone [1]. Note that a Sievert is equal to 1 Gray (Gy) for X-ray and γ-ray exposures, low-linear energy (low-LET) transfer types of radiation, which are the subject of this study.

In 2006, the US National Research Council organized a committee to estimate the Biological Effects of Ionizing Radiation (BEIR). This committee's 7^th^ report, BEIR VII, concluded that the risk of fatal cancer development in a population increases 3–12% per Sievert of low dose or protracted ionizing radiation that the population is exposed to [2]. A four fold difference between the high and low ends of this risk estimate makes it difficult to judge how much effort should be expended to protect society from radiation exposures [3–5].

Notably, annual (non-therapeutic) exposures per person are on the rise due to medical imaging technologies like computed tomography (CT), nuclear medicine, and fluoroscopy. These medical imaging exposures now constitute roughly 50% of the total dose to the population in America. In 1980 they contributed less than 10% of the cumulative US population dose [1,3,6,7].

It is critical to understand the true risks of radiation in order to guide efforts to reduce exposure—especially in light of rising medical exposures.

### Estimating low dose and protracted risk from acute high dose data

**Fig 1:**
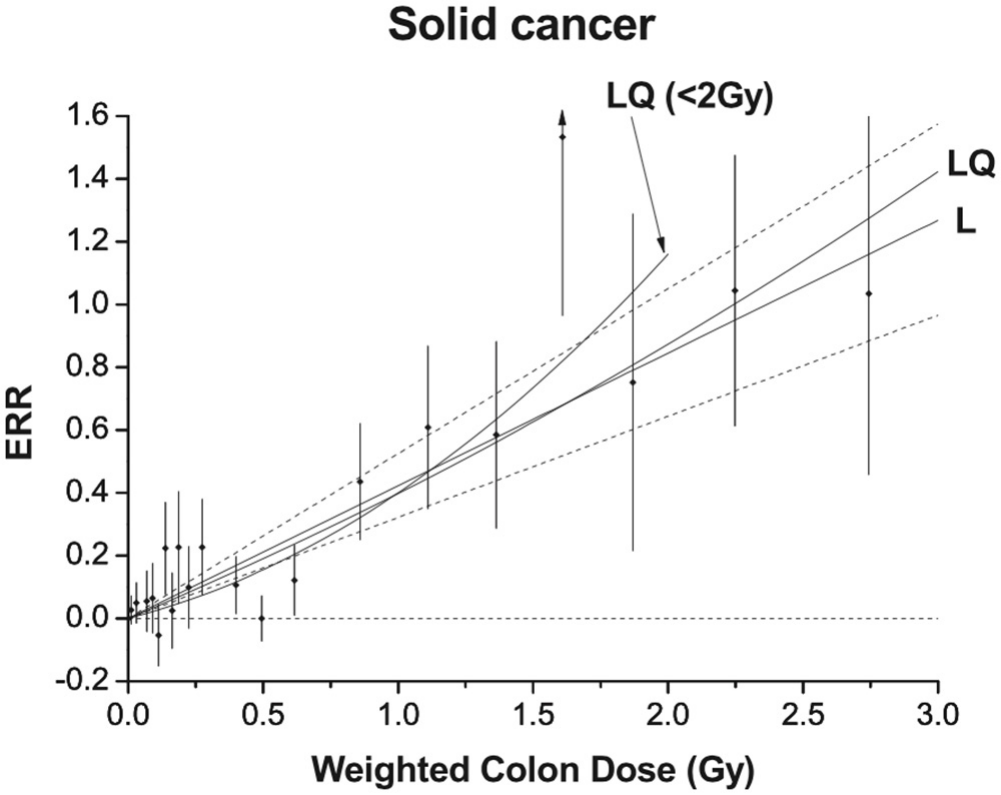
Excess relative risk of solid cancers as a function of dose in atomic bomb survivors. *Reprinted with permission from figure 3 of “Studies of the mortality of atomic bomb survivors*, *Report 14*, *1950–2003*: *an overview of cancer and noncancer diseases*.*”* [8]. Estimated excess relative risk (ERR—equal to relative risk minus one) of solid cancer development vs. mean total colon dose for atomic bomb survivors. These estimates represent the risk of solid cancer development by age 70 to a person exposed at 30 years of age after controlling for the influence of gender and city (Hiroshima vs. Nagasaki) using models specified by Ozasa and others [8]. Black points represent central estimates for each exposure group. Vertical bars represent 95% confidence intervals. Linear (L) and linear-quadratic (LQ) dose response models were both fit to the data and appear as labeled. A linear-quadratic model fit to doses below 2 Gy is shown as well (LQ (<2Gy)). The apparent quadratic component of ERR increase with dose is most pronounced for exposures less than 2 Gy. This curvature is presumably a consequence of the relatively lower than expected solid cancer development risks in the dose range 0.3–0.7 Gy for which neither Ozasa and others nor earlier reports offer an explanation.

The BEIR VII report, like reports from most national and international radiation protection agencies, use data from the lifespan study (LSS) of Japanese atomic bomb survivors as their primary source to estimate cancer risk following radiation exposure. Survivors experienced excess risks of cancer development and mortality that increased with the dose received (Fig 1).

However, most contemporary exposures, such as medical CT-scans, are low-dose exposures, less than 20 mSv, though often delivered acutely at high dose rates [9]. Also, many people receive many small exposures that result in cumulative lifetime exposures at moderate total doses, greater than 100 mSv. The health effects of these low dose and protracted exposures are challenging to estimate. Analysis of atomic bomb survivors are inconclusive because the risk per individual at doses less than 20 mSv is too small to be detected with statistical significance [10] and because all atomic bomb survivors were acutely exposed. Other epidemiological datasets are being developed to explore the risk of protracted exposures, but do not have sufficient statistical power to resolve these questions definitively [11]. Therefore, the health risks of low dose and protracted exposures are currently estimated based on the health consequences observed following acute, often high-dose, exposures and a model of the relationship between dose, risk, and protraction.

The BEIR VII report estimated the risk of low dose and protracted exposures by applying a linear-quadratic dose response model to data from atomic bomb survivors and animal studies as described below. It is our belief, that it is necessary (and possible) to estimate these risks more accurately by using additional exposure data and more appropriate dose-response models.

### BEIR VII used a linear-quadratic dose response model

**Fig 2:**
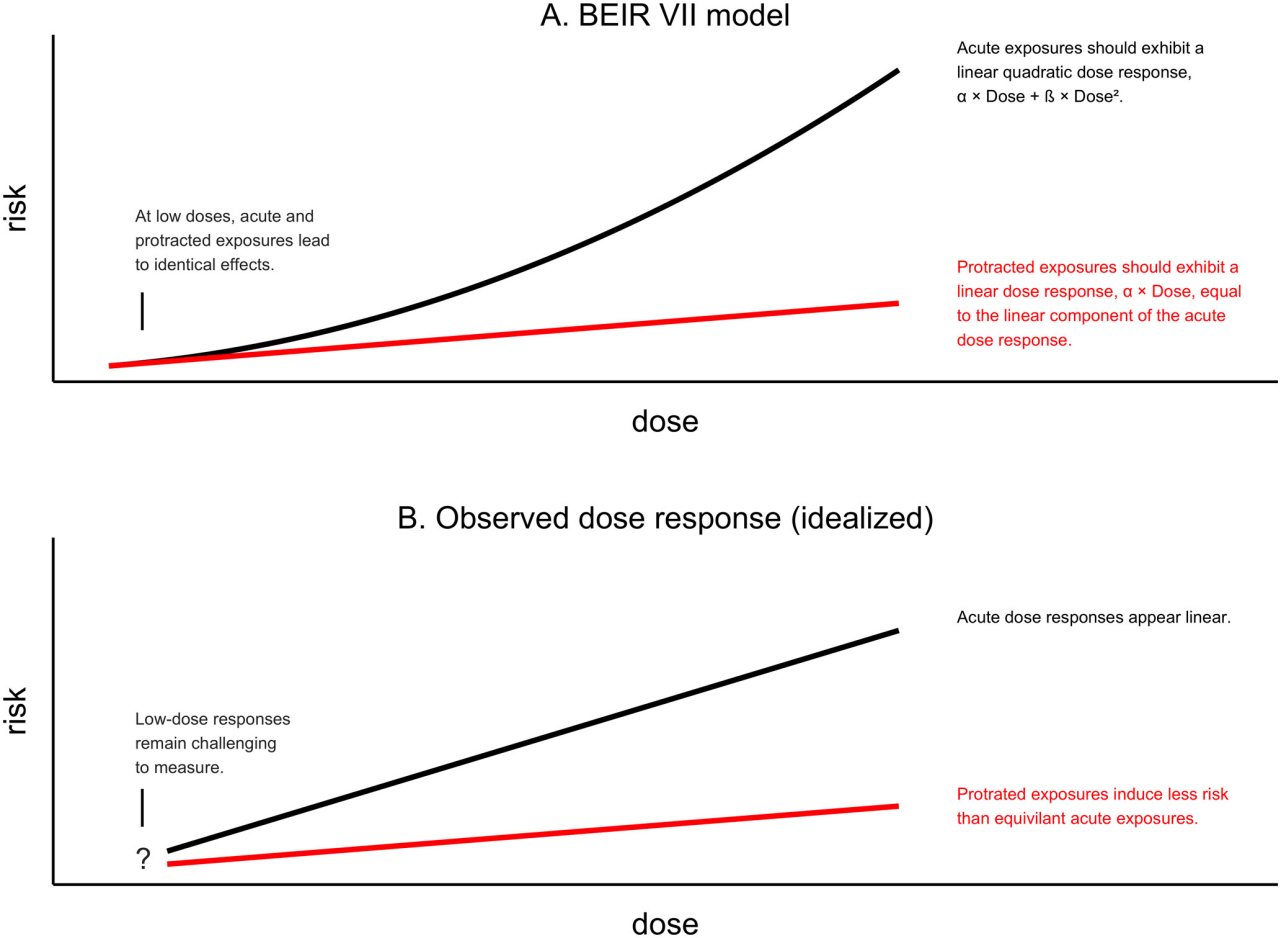
Two possible dose response models based on linear-quadratic model (A) and linear/linear model (B). A schematic representation of a linear-quadratic dose response model, like the one used in the BEIR VII report (A), is shown above an idealized representation of the results of this analysis (B). Each panel shows dose (x-axis) vs. risk (y-axis) in which risk represents the excess risk of carcinogenesis or organism mortality. Black lines represent the response to acute exposures. Red lines represent the response to protracted exposures. Both are applicable to exposures less 1.5 Sv or 2.0 Sv, the maximum doses considered in the BEIR VII report and this one. While the linear-quadratic model predicts that protracted dose-response can be estimated based on the curvature of acute dose response our results show that this is not the case. Also, while the linear-quadratic model predicts that responses to low dose exposure are collinear with responses to protracted exposures, our observations are inconclusive.

To estimate the risk of low dose and protracted exposures, the BEIR VII report employed a linear-quadratic dose response model to estimate DDREF_LSS_ [2]. This model, illustrated in Fig 2A, predicts that the risk of carcinogenesis and mortality following exposures to X-rays or γ-rays in the moderate dose range (below 1.5 or 2 Gy) is the sum of two components, one that increases linearly with dose and another that increases quadratically with dose, where the linear and quadratic coefficients, α and β, are estimated based on observed data. In this work, we challenge the linear-quadratic model with regard to life-shortening and propose use of two separate linear dose response models one for acute/high dose rate exposures and another for protracted/low dose rate exposures (Fig 2B)

The linear-quadratic model assumes that the linear component of risk is unavoidable regardless of the pattern of exposure, while the quadratic component is attenuated at low doses or when an exposure is delivered over sufficient time to allow repair of initial damage before additional damage occurs. Therefore the risk following a low dose, low dose rate, or fractionated exposure is described by only the linear risk component, α, of a corresponding acute exposure.

Concretely, if an exposure is fractionated into distinct equally sized acute exposures separated by enough time for maximum repair then risk is:

$$risk\sim \alpha \cdot dose+\frac{\beta \cdot dose^{2}}{fractions}$$

Where fractions is the number of distinct fractions that a dose has been divided into.

Using the linear-quadratic model, DDREF can be calculated by dividing risk from acute irradiation with the risk of protracted dose exposures.

$$DDREF=\frac{acute\;risk}{protracted\;risk}=\;\frac{\alpha \cdot dose+\beta \cdot dose^{2}}{\alpha \cdot dose}=1+\;\frac{\beta }{\alpha }\cdot dose$$

As shown, DDREF is a function of the ratio between quadratic and linear coefficients, β/α, and dose. As formulated, it can be derived from direct comparisons of protracted and acute exposures. However, because acute exposure risk depends on linear and quadratic terms both, DDREF can also be derived from acute exposure data alone. First by estimating α and β terms based on a quadratic fit to the data and then by extrapolating the risk of protracted exposures from the α term. The more curved an acute dose response is, the higher the DDREF estimate will be.

According to the linear-quadratic model, DDREF depends on dose. The DDREF applicable to atomic bomb survivors, DDREF_LSS_, must be calculated across the range of exposure doses that survivors received. The BEIR VII report showed that DDREF_LSS_ is nearly equivalent to DDREF at 1 Sv [2]. Therefore, DDREF_LSS_ is approximately 1 + β / α. We employ the same approximation throughout this work.

Finally, the complete BEIR VII model includes the notion that there is interference between cancer induction and reproductive cell death (which includes both cell killing and terminal senescence) following acute exposures to doses greater than 1.5 Sv or 2 Sv. Therefore BEIR VII estimated DDREF_LSS_ based only on exposure data less than 1.5 Sv or 2.0 Sv; different cutoffs were used depending on the dataset in question, however a rationale for these particular cutoffs was not provided.

### BEIR VII’s data sources and DDREF_LSS_ estimates

**Fig 3:**
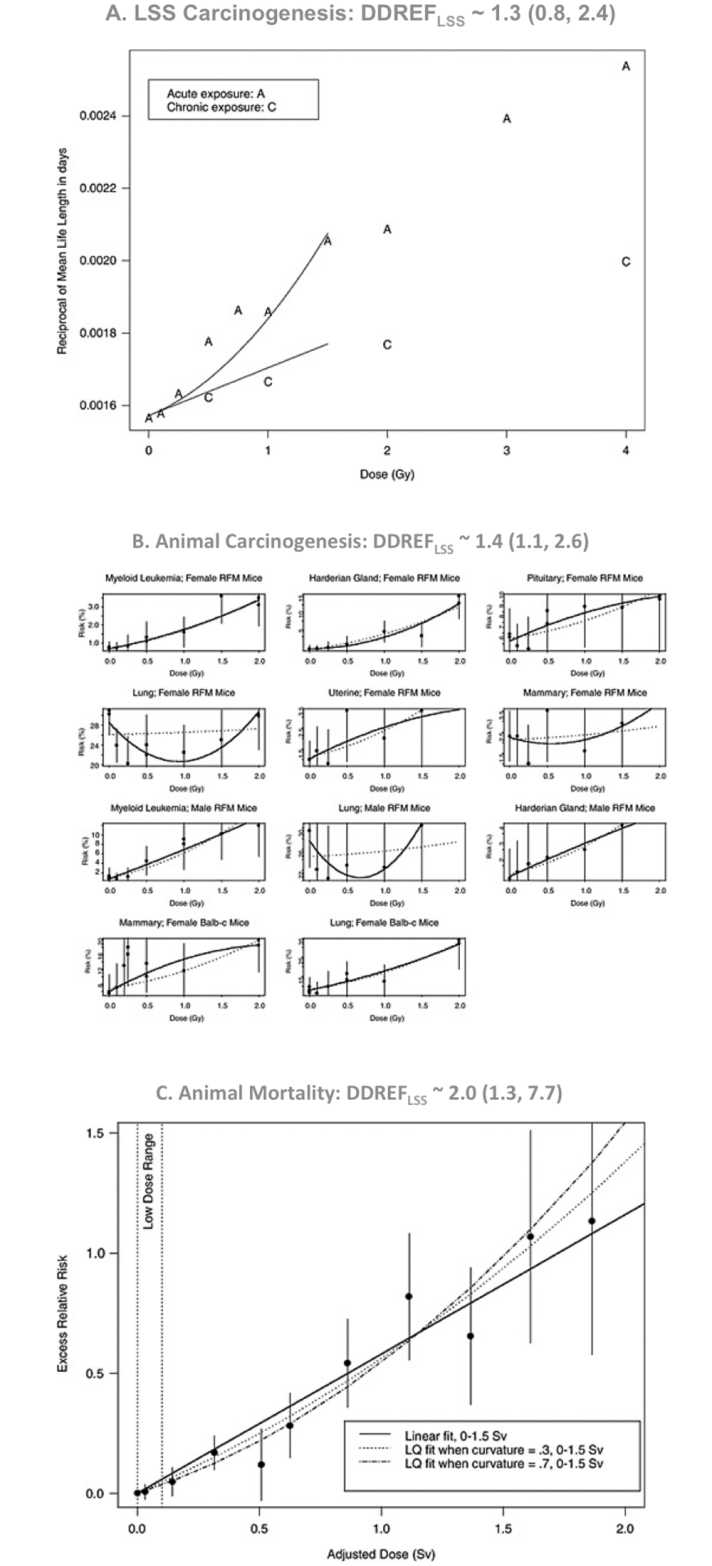
BEIR VII DDREF_LSS_ estimates from 3 data sources. *Reprinted with permission from figures 10–2*, *10B-2*, *and 10B-3 of “Health risks from exposure to low levels of ionizing radiation*: *BEIR VII phase 2”* [2]. Linear-quadratic models used for DDREF_LSS_ evaluation fit to three data sources, (A) excess risk of carcinogenesis in atomic bomb survivors, (B) risk of tumor development in various animal studies, and (C) inverse mean lifespan in two animal studies. All panels show dose (x-axis) vs. various measurements of risk (y-axis). Best-fit linear-quadratic models are shown for each model. The LSS carcinogenesis data shows best-fit models with various curvatures. Animal carcinogenesis data shows best-fit models for each panel individually (solid black lines) and the consensus curvature across all panels (dashed lines). The animal mortality data shows the single best-fit model with both acute (linear-quadratic) and protracted (linear) projections. Only animal mortality data included both acute, “A”, and protracted, “C”, exposures. Above each panel, DDREF_LSS_ estimates derived from the corresponding data source are shown with 95% credible intervals in parenthesis. These estimates were combined (using Bayesian update) to form BEIR VII's central estimate, DDREF_LSS_ ~ 1.5 (1.1, 2.3).

The BEIR VII report fit linear-quadratic dose response models to three distinct data sets: excess cancer incidence rates in atomic bomb survivors exposed to doses less than 1.5 Sv, cancer incidence rates in 11 animal studies with exposures up to 2 Sv, and mortality rates in 2 animal studies with total exposure doses less than 1.5 Sv. Fig 3, reproduced from the BEIR VII report, shows linear-quadratic fit and DDREF_LSS_ estimates for each of these data sets. The profile-likelihood method was used to estimate a different DDREF_LSS_ the relative likelihood of different DDREF_LSS_ values from each data source and Bayesian update was used to combine these separate estimates into one central estimate: DDREF_LSS_ equal to 1.5 with a 95% credible interval from 1.1 to 2.3. Full details of the techniques employed by BEIR VII committee, which we also used in this report, are provided in the methods section.

### Other radiation protection agencies have used a mixture of dose response models

Other national and international agencies, in addition to the BEIR committee, have made their own efforts to estimate the effects of low dose and protracted radiation exposures. Most of their estimates have employed linear-quadratic models to some degree; therefore, our existing work challenges at least some components of their estimates.

The 2006 report from the United Nations Scientific Committee on the Effects of Atomic Radiation (UNSCEAR) used both linear-quadratic and linear-quadratic-exponential (an “S” shaped dose-response) fits to atomic bomb survivor data in order to estimate the risk of a range of exposure doses [12]. These models predict that low dose exposures carry less risk, corresponding to DDREF_LSS_ values of 1.2 to 2.85 (depending on the model and the outcome). However, unlike the BEIR VII report, and our findings, these models do not predict that protracted exposures induce less risk than acute exposures at the same dose. Moreover, we conclude that a linear-quadratic model, like the one UNSCEAR used, is not appropriate for estimating the relative risk of protracted exposures. However, the linear-quadratic-exponential model, that UNSCEAR also employed, might be appropriate. Such a model could explain our observations presented in this work. We discuss this possibility in detail in the discussion.

The National Council on Radiation Protection 1980 report offered an estimate of DDREF_LSS_ from 2 to 10 based on an analysis of animal studies [13]. This report directly compared linear fits to acute exposure data with linear fits to protracted exposure data to estimate DDREF_LSS_. While this technique is the one we recommend based on our current findings, it is important to note that NCRP’s actual analysis had other flaws, for example, the summary DDREF_LSS_ value range, 2–10, was generated informally rather than by a quantitative and reproducible technique.

Finally, the International Commission on Radiological Protection uses a DDREF_LSS_ value of 2. This choice was informed by the work of the NCRP from 1980 detailed above, combined with a linear-quadratic model fitted to atomic bomb survivor data [14].

Ultimately, we believe that all of these dose response estimates have flaws either because they rely too heavily on linear-quadratic dose-response models or because of other problems with their analysis. Notably the UNSCEAR linear-quadratic-exponential model is compatible with results described here. Nevertheless, we recommend caution in any assumed dose-response model and therefore favor comparing linear fits to acute and protracted exposures as in NCRP’s 1980 report.

### The current estimates of contemporary risk should be improved

Several factors make it challenging to measure the risk of contemporary radiation exposures. For example, there is uncertainty in the estimates of the dose that atomic bomb survivors received, in radiation sensitivities of Japanese populations vs. world populations, the value of DDREF_LSS_, and the relative effectiveness of local vs. whole body irradiation. Of these, BEIR VII estimates that uncertainty in the risk of low-dose and protracted exposures is the dominant source of uncertainty in the estimate of the risk of contemporary exposures [2]. A more recent report from UNSCEAR agrees with this conclusion [15].

Recent literature on DDREF_LSS_ highlights these uncertainties. Two recent studies have suggested that BEIR VII’s DDREF_LSS_ estimate may be too high, which would imply that low dose and protracted radiation exposures pose more of a health risk than the current estimates indicate. Jacob and others performed a meta-analysis in 2009 that found that workers exposed to protracted radiation and atomic bomb survivors exposed to acute radiation showed comparable increases in cancer risk for the same total exposure dose [11]. This result, albeit with substantial uncertainty, implies that acute and protracted exposures are equally dangerous, that DDREF_LSS_ is close to 1, and that existing radiation protection standards underestimate the risk of radiation exposure. Similarly, Ozasa and others (2012) suggest that DDREF_LSS_ may be 1 or even less than 1 because, following exposures less than 0.5 Sv, the risk of carcinogenesis in atomic bomb survivors is regularly equal to or greater than the risk suggested by a linear-quadratic or even a linear fit to the data [8]. Based on these studies and other arguments, the German Commission on Radiological Protection (SSK) has recommended that DDREF_LSS_ corrections should not be used to estimate the risks of low dose and protracted exposures [16,17].

On the other hand, Hoel (2015) argues that the DDREF_LSS_ estimate made by the BEIR VII report is too low, and that plausible alterations to the BEIR VII assumptions result in DDREF_LSS_ estimates at or above 2, the number adopted by the International Commission on Radiological Protection (ICRP) [18]. Part of Hoel’s argument is based on the observation by Little in 2008 that an “S” shaped linear-quadratic-exponential dose response describes the atomic bomb survivor data better than a linear-quadratic dose response [19].

Hoel’s observation is an example of a general point. The shape of the dose-response function has a substantial influence over estimates of low dose and protracted exposure risk. Unfortunately, the true shape of the dose-response function for carcinogenesis and mortality is still subject to substantial debate. BEIR VII elected to apply a linear-quadratic model based on the observation that the rate of chromosomal aberrations in cells is linear-quadratic with dose and the assumption that the risk of carcinogenesis (and by consequence mortality) increases linearly with the number of chromosomal breaks that have occurred, the so called linear non-threshold model of cancer induction [2]. This risk model represents only one of the possible approaches to estimate low dose and protracted exposure risks [9]. A variety of alternative models could have been used for the same range of doses, each with distinct implications for the risk of low dose and protracted exposures [20].

Ultimately, the true dose response function is difficult to derive. In part, this is caused by the fact that cellular responses and whole organism responses to radiation exposure are complex. In addition to chromosomal re-arrangements, irradiation of cells leads to other mutations [21], epigenetic changes [22], adaptive effects [23], hypersensitivity [24], and off-target effects [25,26]. Even if the radiation response was completely understood, estimating the carcinogenesis and mortality dose response curves would be difficult because mechanisms underlying these outcomes are also not completely understood [27,28]. Even if the most relevant forms of cellular level damage follow a linear-quadratic dose response it is not certain that the dose-responses of whole organism endpoints such as cancer induction and life-shortening could be described by the same formula.

Notably, even the proponents of the linear-quadratic dose response model, like BEIR VII, limit its use to describing dose-responses under some limit (e.g. 2 Sv), above which it is assumed that cell reproductive death mitigates dose response.

### Additional data discredit the linear-quadratic model (and BEIR VII’s DDREF_LSS_ estimates)

We began our investigations hoping to improve the estimate of DDREF_LSS_ by increasing the pool of data used to estimate it. The animal mortality dataset used for BEIR VII report consisted of 17,322 mice from two studies conducted at Oak Ridge National Laboratory. There have been dozens of other large, lifespan animal studies that have been conducted to estimate the effects of dose and dose protraction. Efforts by the International Radiobiology Archives [29], The European Radiobiology Archives [30], and the Janus Tissue Archives [31,32] have made many of these datasets readily available on the internet. We used the available archived information to establish an expanded animal dataset of 28,289 mice from 16 studies in order to revisit BEIR VII's DDREF_LSS_ estimate with more information (see Table 1).

**Table 1:** Data selection by inclusion criteria.

Studies	Treatments	Animals	Criteria
302	6,810	452,595	All animal data from ERA and Janus archives
124	2,611	205,758	Individual-level animal data available
35	827	116,542	External radiation exposures
35	457	76,096	Low-LET, whole body exposures
34	230	45,730	Total dose equal to or below 1.5 Sv
32	175	43,043	No other treatments (e.g. no chemical exposures)
26	119	34,439	Digitized data on treatment and lifespan confirmed by primary literature
16	91	28,289^a^	At least three distinct treatment groups per stratum so that a linear-quadratic model could be fitted
9	71	20,325^b^	At least three distinct treatment groups after stratifying by study ID

The number of distinct studies, treatment groups, and individual animals that remained eligible for analysis after application of each of the inclusion criteria. Complete definitions of these criteria are elaborated in the methods section.

^a^ dataset used for the “BEIR VII model”, “Hormetic correction”, and “Heterogeneity correction” models.

^b^ a more restricted dataset used in the “Stratification by study” and “Survival analysis” discussed in the results section.

However, rather than establishing a better estimate of DDREF_LSS_, we found that BEIR VII's dose response model did not fit the observed data (Fig 2). Specifically, estimates of DDREF_LSS_ based on the curvature of acute exposure data were low, never significantly greater than 1, implying that protracted and low-dose exposures have a similar risk per Sievert as acute exposures. By contrast, estimates of DDREF_LSS_ based on data that directly compared acute and protracted exposures were infinitely high, implying that low dose exposures are neutral with respect to carcinogenesis or life shortening.

The linear-quadratic dose response model does not predict this contradiction. Both ways of determining DDREF_LSS_ should lead to the same estimate, not two significantly different estimates. Therefore we question the validity of the linear-quadratic model and the DDREF_LSS_ estimate that was derived from it.

We argue that future attempts to estimate the risk of protracted exposures should not assume that these risks can be derived from the apparent curvature of acute responses. Instead, estimates should be based on direct comparisons of acute and protracted exposures.

Ideally, the risk of low dose exposures should also be based on direct comparisons of high-dose and low dose exposures. Unfortunately, statistical considerations make it challenging to conduct this comparison with meaningful precision. The risk of low dose exposures will continue to be extrapolated from the risks observed following high dose exposures. The question of whether these risks are collinear with the risks from protracted exposures, acute exposures, or something else entirely should continue to be debated.

Finally, we encourage the radiation research community to make more data publicly available for analysis. We show in this work how archival data from the European Radiobiological Archives (ERA) and Janus archives can be leveraged to correct existing dose-response models. While progress in data digitizing has been made, these archives are still incomplete. Irradiated animal archives from Japan and former USSR are not presently available through ERA or any other public archive. Inclusion of these data would likely further improve the models used to estimate the risks of contemporary exposures.

## Methods

### Data selection

New data included in this work come from animal irradiation experiments conducted in the United States and Europe and deposited into or connected with the Janus (http://janus.northwestern.edu) or ERA (https://era.bfs.de) databases. These databases are part of an ongoing effort to aggregate and make public all of the data generated in studies of animals exposed to radiation [29]. Most of the material in these archives comes from large, lifespan studies conducted during the cold war.

Data were selected to adhere to the same criteria used in the BEIR VII report so that the results of this study could be directly compared to the results of BEIR VII. Steps were taken to ensure the reliability of archived data by cross validating it against primary literature. Initially, we considered all of the individual level animal data available from the ERA and Janus archives. As in BEIR VII's animal mortality analysis, we selected animals that received whole body, external beam, and low-LET radiation exposure at total doses <1.5 mSv. We excluded animals that received additional non-radiation treatments like hormones or radioprotectors.

Once these data were selected, they were verified against the original literature, to ensure that treatment conditions and mean lifespans matched published results. In several cases data were excluded from the analysis because they were irreparably different than published results. These exclusions and a detailed rationale for each of them are shown in S1 Table.

Finally, data were only included if they contained 3 distinct treatment groups per stratum necessary to fit a linear-quadratic dose response model. The specific stratification criteria are addressed in the next section. The number of animals that were eligible for analysis after applying each selection criterion is shown in Table 1.

Notably only mouse data met all the selection criteria specified. Therefore, even though ERA and Janus databases contain information on rats, dogs, and other species, these data were not included in this analysis, usually because their exposures were greater than BEIR VII’s 1.5 mSv cutoff. It is also important to mention that more data could be added to the pool by digitizing additional data from existing animal experiments, both for the experiments conducted in the US and still not digitized, and for data from experiments conducted in the former USSR. With regard to animal radiation experiments done in Japan, data are generally digitized, but still not available to the public. Finally, it may be possible to find “external data sources” to confirm the treatments posted by Janus and ERA websites by exploring some of the old animal work reports.

### Data stratification

As in the BEIR VII report, data were initially stratified by strain, gender, and age at exposure. Data were not specifically stratified by research institution, but, as a consequence of the chosen stratification conditions, each stratum contained data from only one institution.

The dose response was fit to linear-quadratic models. It is known that radiation sensitivity varies by age, strain, and gender, among other factors. Therefore as in the BEIR VII analysis, the linear, α, and quadratic, β, coefficients were allowed to take on different values within each stratum, while the ratio between quadratic and linear coefficients, β/α, was constrained across all strata. In this way all strata could contribute to one central DDREF_LSS_ estimate despite variations between groups (e.g. if different genders or different mouse strains have different linear and quadratic coefficients).

Finally, one analysis was performed wherein data were additionally stratified by study to determine if the results remained consistent without the assumption of between-study comparability.

### BEIR VII linear-quadratic model

We applied the linear-quadratic model as developed by the BEIR VII report to lifespan data. Concretely:

$$\frac{1}{mean\left(lifespan\right)}=\alpha_{stratum}\cdot dose+\frac{\beta_{stratum}\cdot dose^{2}}{fractions}+intercept_{stratum}+\epsilon$$

Where lifespan, dose, number of fractions and the residual, ɛ, took on distinct values for each treatment group and quadratic, β, linear, α, and intercept coefficients were determined separately for each stratum. The β/α ratio was fixed across all strata. Concretely:

$$\frac{\beta_{stratum}}{\alpha_{stratum}}=\theta$$

Where θ took on a single value for the entire data set. A range of β/α ratios from -1 to infinity, corresponding to DDREF_LSS_ from 0 to infinity, were considered to establish 95% credible intervals as described below. Likelihood was estimated for each ratio using the ordinary least squares maximum likelihood estimator, assuming normal error distribution, and weighted by inverse variance in lifespan for each treatment group. Concretely:

$$\text{log}\left(likelihood\right)=-\frac{n}{2}\left(log\left(\frac{2\pi }{n}\right)+\sum +\text{log}\left(\sum w\epsilon^{2}\right)+1\right)$$

Where v is the variance in the outcome, n is the number of treatment groups, and ɛ is the residual difference between observed outcomes and those predicted by the model.

### Credible intervals for β/α ratios

Likelihood was estimated for each β/α ratio as noted in the sections describing each particular model. A 95% credible interval was determined by the profile likelihood method [33]. Concretely, the 95% credible interval consists of all β/α ratios tested with likelihoods within 1.92 fold of the likelihood for the optimal β/α ratio.

### Conversion between β/α and DDREF_LSS_

Using the linear-quadratic model DDREF = 1 + (β/α) ⋅ dose. As in the BEIR VII model, DDREF at 1 Gy, DDREF_1 Gy_ was used to approximate DDREF_LSS_. Therefore DDREF_LSS_ = 1 + β/α.

### Alternative models

Several adjustments to the BEIR VII linear-quadratic model were also considered as discussed in the results section. These are elaborated below.

#### Eliminating the hormetic paradox

Coefficients were restricted to positive values to avoid the contribution of hormetic-type observations. The justification for this choice is detailed in the results section. Concretely:

$$\begin{array}{l} \alpha_{stratum}>0\\ \beta_{stratum}>0 \end{array}$$

This had the effect of preventing hormetic-type observations from contributing to the DDREF_LSS_ estimate.

#### Accounting for heterogeneity

First meta-regression was performed using a fixed effects model. This regression had the same form as the BEIR VII linear-quadratic model with positive constraints on β and α as described above. Likelihood was calculated, also as described above.

Next, the DerSimonian Laird approach was used to estimate random effects variation between treatments groups, τ^2^ [34,35]. Concretely:

$$\tau^{2}=\frac{\sum {\left(\frac{\epsilon }{\sigma }\right)}^{2}-\left(n-df\right)}{\sum \left(\sigma^{2}-extract\_diagonal\left(V^{-1}X{\left(X^{T}V^{-1}X\right)}^{-1}X^{T}V^{-1}\right)\right)}$$

Where ɛ is the difference between observed outcomes and the predictions of the model, σ is the measured standard deviation of inverse mortality in each treatment group, n is the number of treatment groups, df is the number of features in the model, X is a matrix of model features, extract_diagonal extracts the diagonal component of a matrix into a vector, and V is the diagonal matrix defined by σ^2^.

Finally, the variance estimate for each treatment groups was adjusted to account for the random effects estimate. Concretely, the new variance estimates equal σ^2^ plus τ^2^. The BEIR VII linear-quadratic model was rerun. Likelihood was calculated as shown above with updated variance and weight estimates. This analysis was performed using the metaphor library in R [36].

#### Stratification by study

Study was added to the stratification criteria in addition to strain, sex, and age at exposure. As before, a minimum of 3 treatment groups per strata was required for inclusion in the analysis. This requirement eliminated many groups of animals from analysis as shown in Table 1. In all other respects this analysis was the same as the meta-regression analysis described in the previous section.

#### Survival analysis

Mortality over time was modeled by fitting a Cox proportional hazards model [37] with a linear-quadratic dose response:

$$\lambda_{stratum}\left(t\right)=\lambda_{stratum}\left(t\right)e^{{}_{{}^{\alpha }stratum}\left(dose+\theta \frac{dose^{2}}{fractions}+Z\right)}$$

Where λ_stratum_(t) is the hazard rate over time for a particular stratum, the rate at which animals are expected to die at any time, t. As before, the linear coefficient for each stratum, α_stratum_, was restricted to positive values. Z is an estimate of the random distribution of organism mortality rate by group estimated as described above. All other factors are the same as in previous models. This analysis was performed using the coxme library in R [38].

### Tools and scripts

The scripts used to perform these analyses are available in the Janus github repository, https://github.com/benjaminhaley/janus. Data from the ERA and Janus archives was consolidated and validated using https://github.com/benjaminhaley/janus/blob/master/scripts/exp/radiation.R. The resulting data, used in this analysis, is in https://github.com/benjaminhaley/janus/blob/master/data/external5.rds. This data was filtered and analyzed using https://github.com/benjaminhaley/janus/blob/master/scripts/exp/ddref.Rmd. Ongoing analyses are live online at http://rpubs.com/benjaminhaley/ddref.

The analysis was performed in R [39] using plyr [40], dplyr [41], ggplot2 [42], survival [43], metafor [36], reshape2 [44], xtable [45], pander [46], lme4 [47], and coxme [38] libraries.

## Results

### The expanded animal data set: Included data

28,289 mice in 91 treatment groups from 16 studies were selected for analysis. Inclusion criteria were based on the BEIR VII analysis and are detailed in Table 1. In one of the four analyses conducted, the data were further restricted so that mice were only directly compared if they belonged to the same study. This reduced the size of the data set to 20,325 mice in 71 treatment groups as depicted in the last row of Table 1.

**Fig 4:**
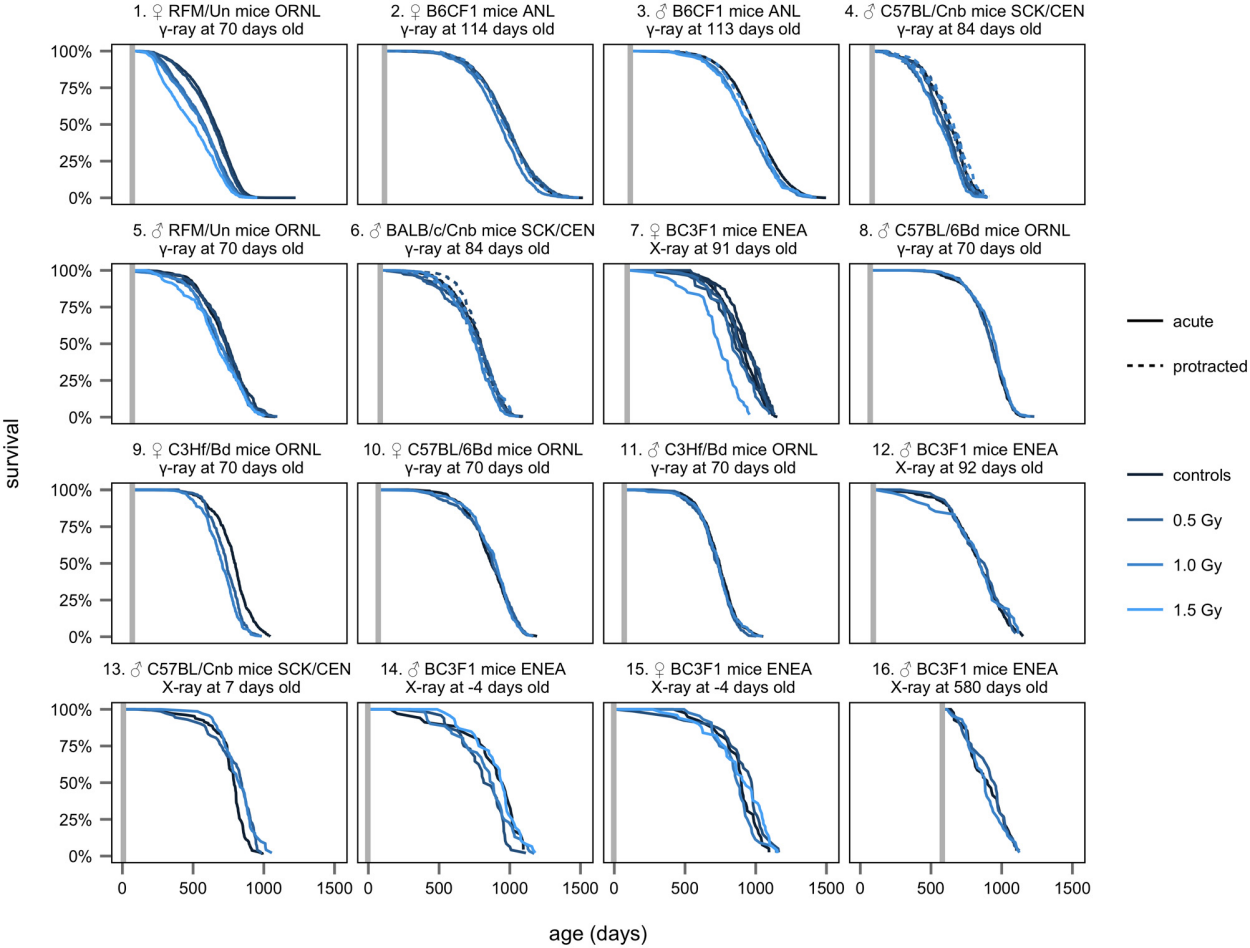
Survival vs. dose. Kaplan-Meier survival curves based on individual animal data show the percent of surviving animals vs. age for each treatment group of the expanded animal data set used in this analysis. The color of each curve indicates total dose, from dark blue (unexposed controls) to light blue (up to 1.5 Gy). A solid line indicates acute exposures. A dashed line indicates fractionated exposures. A vertical gray line indicates age at first exposure. Treatments are stratified by sex, strain, type of radiation, and age at first exposure as labeled. The strata are presented in order of total number of animals included, so that the 1st strata on the top left has the most animals, 6977, and the 16th strata on the bottom right has the least, 126. This same ordering is maintained in all subsequent figures, as are the strata identifiers (e.g. strata labeled #2 always shows data from female B6CF1 ANL animals, 114 days old at the time of first exposure). Please note that the bottom row contains data from studies that investigated the effects of radiation exposure on very young (pre-natal and neonatal) and very old mice. In addition, note that the uppermost leftmost stratum contains data used in the original BEIR VII analysis. This is only the acute exposure data from that analysis, as the data from protracted exposures was not available for individual mice.

The survival curves for the animals that were selected for analysis are shown in Fig 4. The study design and lifespan of each treatment group is shown in S2 Table.

### Re-estimate of DDREF_LSS_ using the BEIR VII’s linear-quadratic method

As in the BEIR VII report, we fit linear-quadratic models to lifespan data according to the function:

$$mortality\;\sim \;\alpha \cdot dose+\frac{\beta \cdot dose^{2}}{fractions}$$

Again, as in the BEIR VII analysis, inverse mean lifespan was used as a proxy for animal mortality. Linear and quadratic coefficients, α and β, were determined for each analysis stratum. Mice within each stratum were of the same strain, sex, and age at exposure—factors that are widely known to affect radiation sensitivity, and therefore linear and quadratic coefficients values [48]. While the values of α and β were allowed to vary by stratum to reflect varying radiation sensitivity, the ratio between quadratic and linear coefficients, β/α, was fixed across all strata of the data in order to find a single, central, DDREF_LSS_ estimate. A range of β/α ratios, corresponding to DDREF_LSS_ from zero to infinity, were fit to the data and the likelihood of each ratio was found in order to establish a 95% confidence interval by the profile likelihood method (see methods section for full details). A 'fraction' was any dose delivered in 1 hour or less.

**Fig 5:**
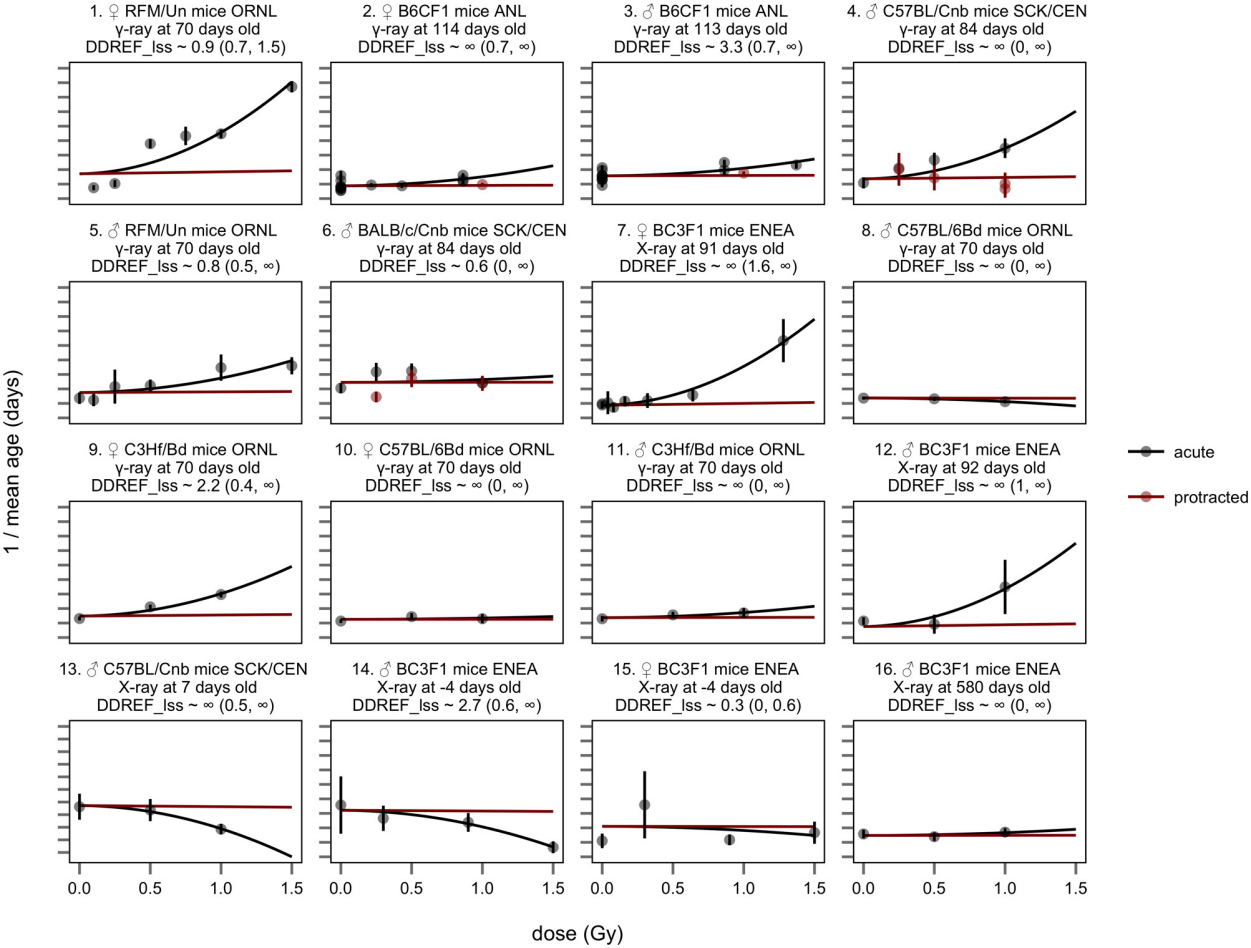
BEIR VII model applied to expanded animal data set. The relationship between inverse mean lifespan, y-axis, and dose, x-axis is shown grouped by analysis strata. Strata are organized and labeled as in Fig 4. The y-axis maintains a constant scale, 0.0001 days per tick with a different baseline for each stratum. Single points indicate results for each treatment group with standard error bars as indicated. Acute exposures and quadratic dose response estimates are shown in black, protracted exposures and linear dose response estimates are shown in red. Please note that protracted exposure data was available in only few cases—strata 2, 3, 4, and 6. DDREF_LSS_ estimates from each stratum analyzed independently are listed in each facet label with 95% credible intervals in parentheses. These are also shown in S3 Table. The central DDREF_LSS_ estimated from the full data set is infinite with a 95% confidence interval from 2.9 to infinity (see Table 2).

The results of these fits are shown in Fig 5. Using the BEIR VII method, DDREF_LSS_ was estimated to be infinite with a 95% credible interval from 2.9 to infinity. An infinite value of DDREF_LSS_ would imply that the α term is zero and that protracted exposures have no effect on lifespan. This DDREF_LSS_ estimate, greater than 2.9, would suggest that BEIR VII’s existing DDREF_LSS_ estimate is likely too low. However, we do not believe that this argument is valid because it ignores obvious problems with the fit of this model to the data as discussed in the next section.

### Acute data vs. acute-protracted comparisons

The linear-quadratic model does not fit all of the exposure data well in Fig 5. For example the first stratum shows the results from the largest study included in this analysis, animals acutely exposed at Oak Ridge National Laboratory. These data do not fit the quadratic curve that is applied to them (solid black line), and instead appear approximately linear. Similar arguments can be applied to the acute dose-responses in strata 4 and 5. It appears from the figures that these acute dose-responses are closer to linearity than can be accommodated by the linear-quadratic model.

**Fig 6:**
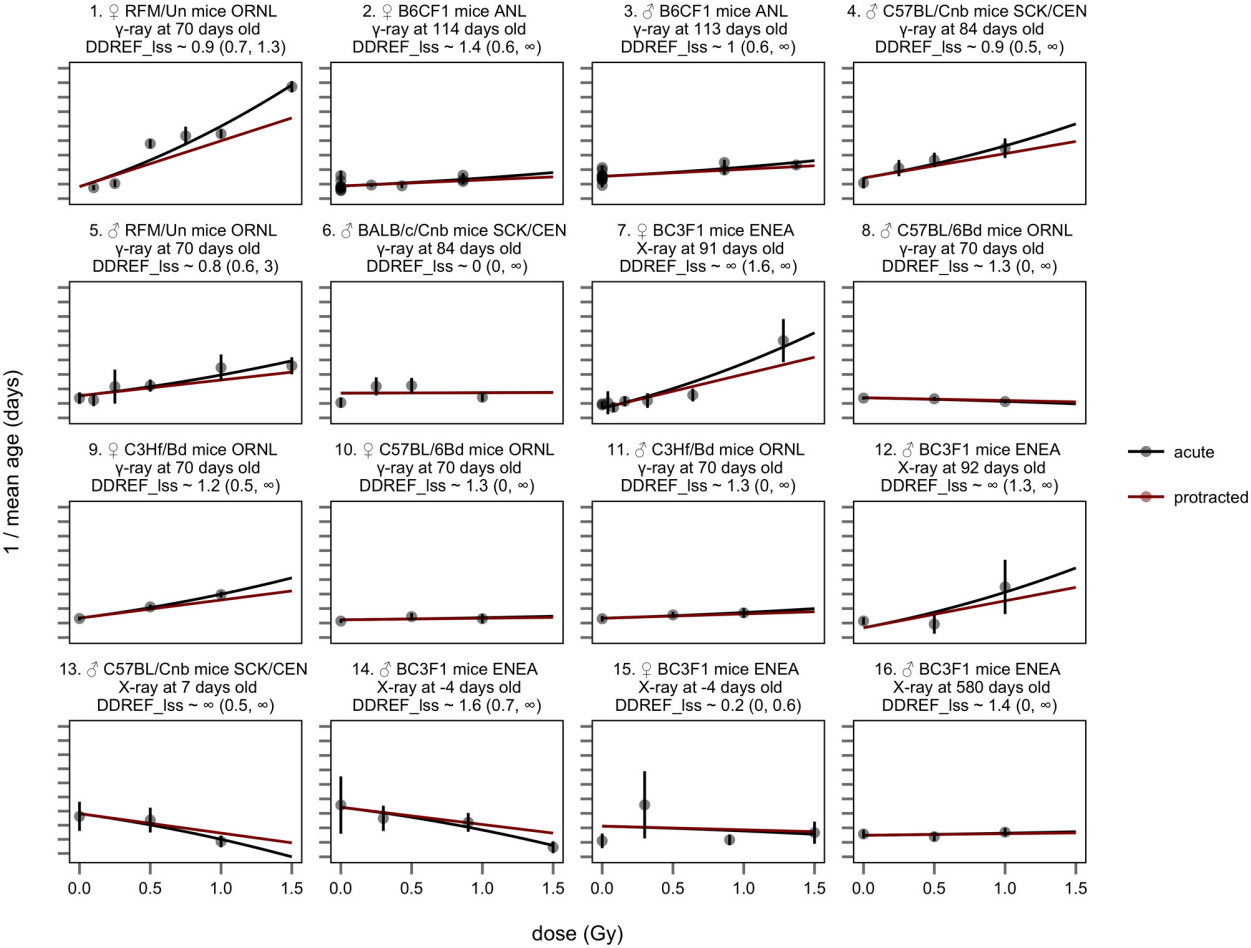
BEIR VII model applied to acute exposures only. Identical to Fig 5, except that data are restricted to animals that received only acute radiation exposures. Protracted extrapolations, estimated from the linear term of acute exposures, are still shown (red lines). Notably, protracted extrapolations are very similar to acute risk estimates because these dose-responses are nearly linear with only a minimal quadratic curvature. This analysis is similar to BEIR VII's estimates of DDREF_LSS_ based on atomic bomb survivors and animal carcinogenesis data that only included acute exposure data as well (Fig 3).

**Fig 7:**
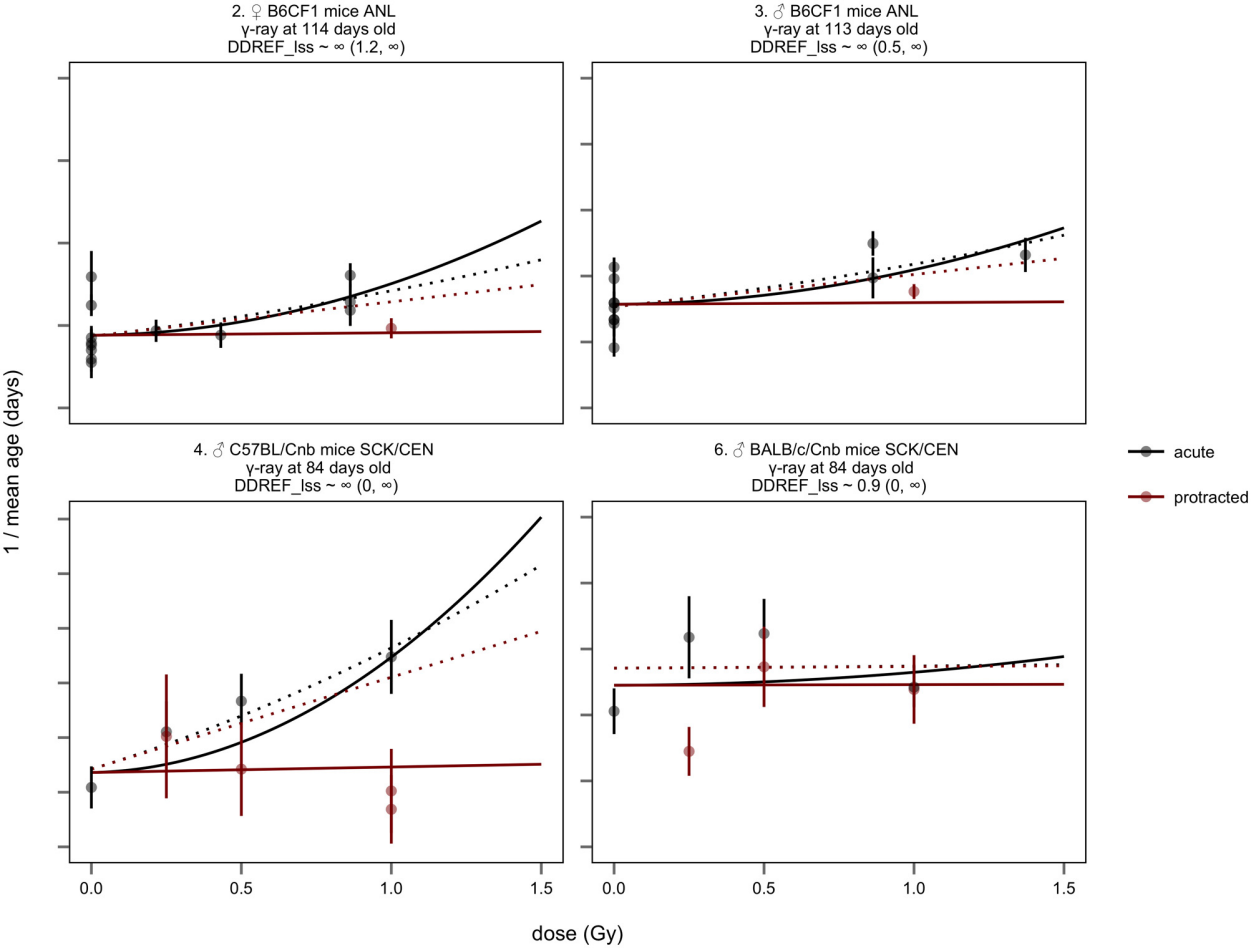
BEIR VII model applied only to protracted-acute comparisons. Similar to Fig 5, except that data are restricted to strata that received both acute and protracted exposures. Also, in contrast to Fig 5, fits based on acute data alone (the same as those in Fig 6) are shown as dotted lines for comparison. Note that the real risk of protracted exposure is substantially lower than the risk projected based on acute data. Also, note stratum 4 has two protracted exposures at 1.0 Gy corresponding to two different fractionation patterns as described in S2 Table.

To test this supposition, we fit BEIR VII’s linear-quadratic dose response model to two subsets of the data independently—in one instance we applied the model to data from acutely exposed animals in each strata, extracting both linear and quadratic coefficients from acute data (Fig 6), in the other, we applied the model only to data from strata that included both acute and protracted exposures, and calculated linear and quadratic coefficients based on both exposure types (Fig 7). The linear-quadratic model predicts that these two subsets should lead to similar DDREF_LSS_ estimates. Instead they are significantly different.

The results of the two separate fits confirmed our qualitative observations. When linear-quadratic models were fit to acute exposure data (Fig 6), DDREF_LSS_ was estimated to be low, 1.3 with a 95% credible interval from 0.9 to 3.0. When data were restricted to direct comparisons of acute and protracted exposures (Fig 7), DDREF_LSS_ was estimated to be infinite with a 95% credible interval from 4.8 to infinity, significantly higher than the estimate based only on the curvature in acute dose response (p < 0.01).

When the linear-quadratic model is applied to acute exposure data it overestimates the risk of protracted exposures. This is highlighted in Fig 7 where dotted lines show the estimates of dose-response based only on acute data. These estimates fit the acute exposure data reasonably well, but overestimate the risk of protracted exposures. This failure of the linear-quadratic model to fit the observed data calls into question not only existing DDREF_LSS_ estimates, but also the conceptual basis used to estimate the relative risk of low dose and protracted exposures as elaborated in the discussion of this paper.

### Variations on BEIR VII’s linear-quadratic model

There are many plausible alternatives to the methodological assumptions made by the BEIR VII report. For example, one can argue that animals from distinct studies should not be combined into a single analysis stratum. Perhaps treatment conditions improved between two studies leading to an increase in longevity that was unrelated to changes in radiation exposure.

We wanted to be sure that our results were consistent, even if the data were analyzed with an alternative, but still plausible, formulation of the linear-quadratic dose response model. Therefore, we re-analyzed the data using several variations on the original BEIR VII methodology. We applied each variation to the full dataset like the analysis depicted in Fig 5. We also applied each variation to the subsets of the data described above, one consisting only of acute exposures (Fig 6) and the other consisting only of strata that included both acute and protracted exposure data (Fig 7). DDREF_LSS_ estimates from these variations are shown in Table 2 and discussed in the sections below.

**Table 2:** DDREF_LSS_ estimates for various models.

Model	All data	Acute data	Comparison data
*BEIR VII model*	∞ (2.9, ∞)	1.3 (0.9, 3.0)	∞ (4.8, ∞)
*Hormetic correction*	∞ (2.3, ∞)	1.2 (0.9, 3.4)	∞ (4.8, ∞)
*Heterogeneity correction*	1.3 (0.9, 5.5)	0.9 (0.8, 1.3)	∞ (2.0, ∞)
*Stratification by study*	1.0 (0.8, 1.6)	1.0 (0.8, 1.2)	∞ (2.2, ∞)
*Survival analysis*	4.8 (1.5, ∞)	0.9 (0.7, 1.5)	∞ (2.5, ∞)

DDREF_LSS_ estimates for a variety of models. Central estimates are shown with 95% credible intervals in parentheses. Each model in this report, described in detail in the text, was applied to three different divisions of the data to produce three different DDREF_LSS_ estimates as listed the three rightmost columns. “All data” refers to DDREF_LSS_ estimates based on all of the available data (as in Fig 5). “Acute data” refers to DDREF_LSS_ estimates based only on the apparent curvature of acute exposure data in each stratum, excluding protracted exposure data (as in Fig 6). “Comparison data” refers to DDREF_LSS_ estimates based on strata that included both acute and protracted exposures, excluding strata that only included acute exposures (as in Fig 7). Note that the estimates of DDREF_LSS_ in this study from data that compares acute and protracted exposures are always significantly larger than the estimates based on acute exposure data alone. Moreover, the estimates based on comparisons of acute and protracted exposures are also significantly larger than the central estimate of DDREF_LSS_ from the BEIR VII report (see Table 3).

**Table 3:** DDREF_LSS_ estimate from the original BEIR VII report.

Model	Central estimate	Acute atomic bomb survivor data	Acute animal carcinogenesis data	Comparison animal mortality data
*Original BEIR VII analysis*	1.5 (1.1, 2.3)	1.3 (0.8, 2.4)	1.4 (1.1, 2.6)	2.0 (1.3, 7.7)

DDREF_LSS_ estimates from the original BEIR VII report. Central estimates are shown in the first column alongside the sub-estimates from various datasets that were used to develop these central estimates. 95% credible intervals are shown in parenthesis.

Regardless of the model variations, we found that the linear-quadratic model never fit the data well. Concretely, DDREF_LSS_ estimates based on curvature in acute exposure data were consistently low, never significantly greater than 1 (Table 2 “acute data”). DDREF_LSS_ estimates based on data from strata that directly compared acute and protracted exposures were consistently high, always significantly higher than 1 and always significantly higher than the corresponding estimates based on only acute exposure data (Table 2 “comparison data”). These results all contradicted the assumptions of the linear-quadratic model that predicts that these two estimates should lead to the same value.

Central estimates of DDREF_LSS_, based on all of the available data, varied substantially between methodologies and were not consistent when compared to each other (Table 2 “all data”). This is not surprising given the poor fit that linear-quadratic models have to the data.

#### Eliminating the hormetic paradox

**Fig 8:**
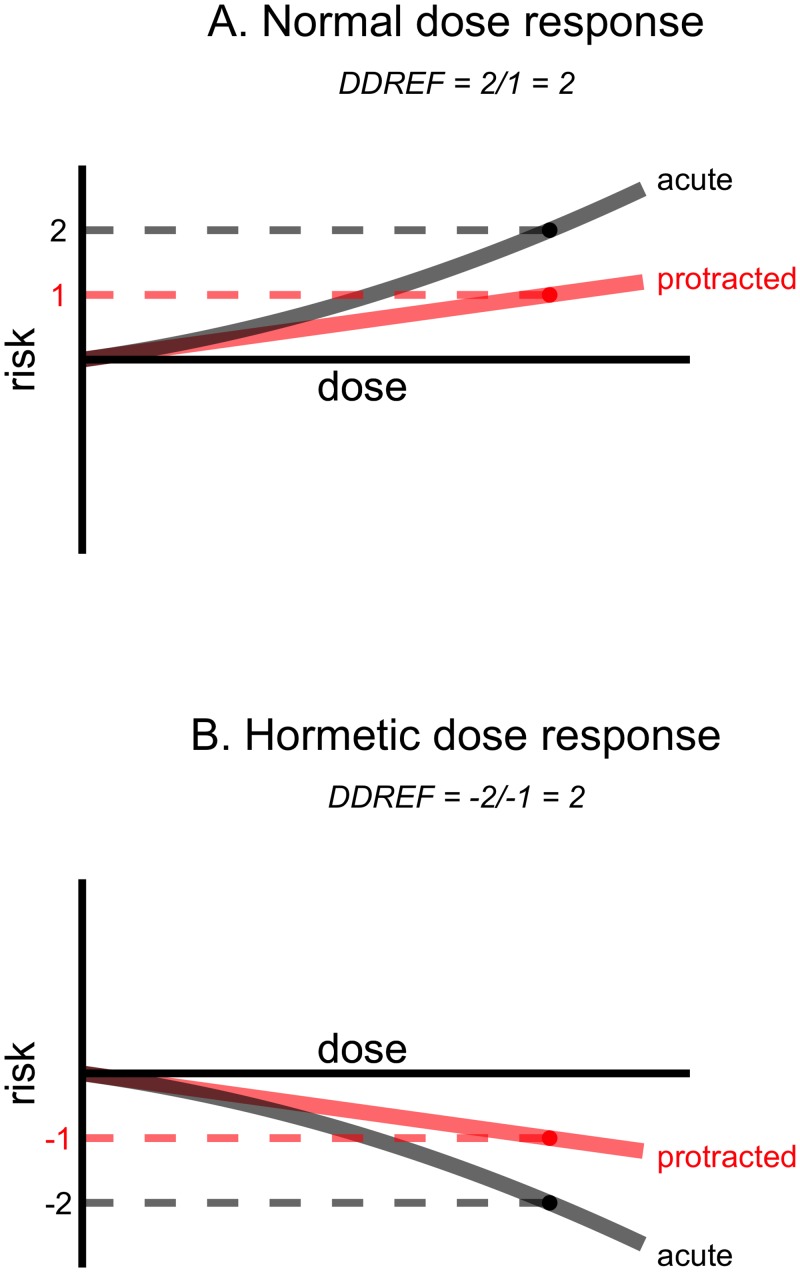
The hormetic paradox. The same DDREF estimate, 2 in this example, can be obtained when (A) acute exposures appear more damaging than protracted exposures or when (B) acute exposures appear more beneficial than protracted exposures.

As shown in Fig 5, several smaller strata (8, 10, 13, 14, and 15) had apparently hormetic responses. Exposed animals lived longer than comparable controls. The concept of DDREF is paradoxical when applied to data that shows a pattern of hormesis. When an exposure is deleterious, a high DDREF indicates that protracted exposure is less *deleterious*. When an exposure is beneficial a high DDREF indicates that protracted exposure is less *beneficial*. Therefore, one single DDREF value could simultaneously predict that protraction leads to more or less carcinogenesis depending on whether acute exposures induce a hormetic-type or deleterious response. This paradox is illustrated in Fig 8.

Should these studies contribute to the central DDREF_LSS_ estimate?

It can be argued that they should not, because:

1. DDREF_LSS_ is applied to atomic bomb survivors who had deleterious, not hormetic-type, dose responses.
2. The linear-quadratic model, with deleterious chromosomal aberrations as its mechanistic foundation, does not predict that hormetic effects should be possible.
3. Hormetic responses were only observed in a minority of smaller animal studies.

Therefore we opted to limit the linear, α, and quadratic, β, coefficients to positive values in this and subsequent analyses. This had the effect of eliminating data that showed hormetic-type dose responses from contributing to the DDREF_LSS_ estimate.

DDREF_LSS_ estimates following this 'hormetic correction' were similar to the results based on the BEIR VII model as shown in the first row of Table 2.

#### Accounting for heterogeneity among treatment groups

BEIR VII weighted their regression analysis by the variance of the mean lifespan observed in each treatment group. This approach neglects random effects—systematic sources of variance that affect entire treatment groups regardless of their size. For example, entire treatment groups may have been subject to variations in animal living conditions or radiation exposures. These variations are not apparent when individual treatment groups are analyzed in isolation. Rather, they become apparent when multiple treatment groups are compared to each other.

A random effects model estimates unmeasured variance between treatment groups, and adds it to the measured variance within each treatment group. This results in a more balanced weighting (with respect to possibility of existence of errors) between large and small treatment groups.

We applied the DerSimonian Laird method [34] to estimate random effects and increase the variance estimates for individual treatment groups in this and subsequent models. Details of this adjustment are described in the methods section.

Evidence of random effects was present in each analysis (p < 0.01). Accounting for heterogeneity had the effect of lowering the DDREF_LSS_ estimates as shown in Table 2. Nevertheless, as before, DDREF_LSS_ estimates based on acute data remained significantly lower than estimates limited to strata that compared acute and protracted exposures.

#### Stratification by study

Like the BEIR VII analysis, our initial analysis combined the results of multiple studies into the same analysis strata. This ignores the possibility that animal mortality and radiation sensitivity varied between studies due to differences in study protocol unrelated to radiation treatment.

Lifespan and radiation sensitivity are affected by cage crowding, pathogen environment, and even ambient temperature [48]. Because these factors often change between studies, it may be prudent to avoid a direct comparison between treatment groups in separate studies. Therefore, we added study origin to the existing stratification conditions, strain, sex, and age at exposure. As before, strata were excluded from the analysis if they had less than the three distinct treatment groups needed to fit a linear-quadratic model.

This exclusion eliminated an additional ~8000 animals (mostly controls) from the analysis as shown in Table 1 and lowered the overall DDREF_LSS_ estimates as shown in Table 2. However, DDREF_LSS_ estimates based on the apparent curvature in acute data remained significantly lower than DDREF_LSS_ estimates based on strata that included a direct comparison of acute and protracted exposures, continuing to violate the linear-quadratic assumptions.

#### Survival analysis

The BEIR VII report used inverse mean lifespan as a proxy for mortality [2]. This is a valid approximation if animal mortality rates increase exponentially with time, as in Gompertz law of mortality [49]. BEIR VII was obliged to assume a theoretical relationship between mortality and age because they did not have access to individual level animal data needed to measure mortality rates directly. In all of the analyses described above, we used the same approach as BEIR VII and extracted inverse mean lifespan values from the individual animal data (see S2 Table) and used these values for DDREF_LSS_ calculations shown in Table 2.

However, we did have access to individual level data and so we ran another analysis using this lifespan data directly (see “Survival analysis” in Table 2). Using the individual level animal data we were able to model excess mortality using observed mortality rates. Therefore, in our final analysis, which we named “survival analysis correction”, we used Cox proportional hazards modeling to fit the change in mortality hazard as a function of age [37].

This modification had the effect of raising the central DDREF_LSS_ estimate relative to the previous analyses. Nevertheless, as in all of the other cases, DDREF_LSS_ estimates based on curvature in acute dose response remained significantly lower than DDREF_LSS_ estimates of strata that directly compared acute and protracted exposures. Therefore in all tested variations, the data continued to violate the assumptions of the linear-quadratic model.

## Discussion

### The linear-quadratic model does not fit the observed data

If dose-response were linear-quadratic then DDREF_LSS_ could be estimated from two types of exposure data: acute radiation exposures or comparisons between acute and protracted radiation exposures. According to the model, both of these data sets should lead to the same DDREF_LSS_ estimate. We find that they do not.

The value of DDREF_LSS_ estimated from the curvature observed in acute exposure data was significantly lower than the value of DDREF_LSS_ estimated from data that directly compared acute and protracted exposures (Table 2). This contradiction is not compatible with the linear-quadratic model and it suggests that a new model is required to estimate the risk of low dose and protracted exposures.

This contradiction between DDREF_LSS_ estimated based on acute exposure data vs. acute-protracted comparison data is also apparent in the original BEIR VII analysis (Fig 3). Animal carcinogenesis and atomic bomb survivor data only included acute exposures and led to relatively low DDREF_LSS_ estimates of 1.3 and 1.4. Animal mortality data were the only data source that directly compared acute and protracted exposures and it led to the highest DDREF_LSS_ estimate, 2.0. While this difference was not statistically significant, it is inline with our current findings.

The contradiction we have discovered calls into question BEIR VII’s DDREF_LSS_ estimate and any DDREF_LSS_ estimates based on a linear-quadratic dose response model. Our results show that the linear-quadratic model is likely to overestimate the risk of protracted exposures. More importantly, our results call into question the theoretical basis of the existing dose response model. If dose-response is not linear-quadratic, then what is it? What implications does the true dose response function have for the risk of low dose exposures? Are the risks per Gy of acute low dose exposures more similar to high dose acute exposures or protracted ones? New models will be needed to answer these questions.

### What is the correct dose response model?

We have shown that the linear-quadratic model employed by the BEIR VII report does not fit the observed data. Specifically, the reduced risk of protracted exposures cannot be extrapolated from the curvature in dose response following acute exposures, because there is no apparent curvature in these data (Fig 2B). The data do not conform to a linear-quadratic model. Which mechanism could explain the observed dose response?

One possibility is that cell reproductive death, assumed to attenuate the dose response by the BEIR VII report, is effective at doses less than 1.5 Sv. This is plausible: At 1.5 Sv ~10% to 50% of cells succumb to reproductive death [50]. Moreover, Little 2008 has shown that the linear-quadratic exponential dose-response, an “S” shaped curve that is expected to result from reproductive cell death, fits atomic bomb survivor data (slightly) better than quadratic models and this fit would imply substantial reproductive cell death (~50%) at 1.5 Sv [19]. If this hypothesis were true, then acute responses would be “S” shaped, even in the range from 0 to 1.5 Sv, and a quadratic fit could appear approximately linear even if dose response curved upwards at the lowest doses. In this case DDREF_LSS_ would be greater than existing estimates, and, correspondingly, the risk of protracted exposures would be lower than existing estimates.

Another possibility is that protracted exposures were safer because the exposed animals were older than their acutely irradiated counterparts because their exposure was extended over a period of time. It is known that radiation sensitivity decreases with age. For example the risk of solid tumor development decreases with age at exposure in atomic bomb survivors by about 10% to 30% per decade [8]. Most radiation studies, including the ones in this analysis, delivered acute exposures at the same time as the first exposure in a fractionated series. Subsequent fractions were delivered to older animals that could be more radio-resistant. This is particularly relevant to animals that received protracted exposures in strata 2 and 3 who aged 420 days between their first and last exposures (S2 Table and Fig 7). However, while age at exposure reduces dose response it does not eliminate it, and could not account for the fact that these studies indicated that DDREF_LSS_ was most likely to be infinite and protracted exposures risk free. Moreover, animals exposed to protracted irradiation in strata 4 and 6 aged at most 10 days before first and last exposure, yet still lived longer than acutely exposed animals in those strata. Ultimately, more data is needed to estimate whether and how much the effects of protraction are driven by differences in age at exposure.

There are many more possible explanations for the observed dose response pattern as well. The effects of radiation exposure at the cellular level are complex. In addition to chromosomal rearrangements, ionizing radiation exposure leads to epigenetic changes [22], adaptive responses [23], hypersensitivity [24], and off-target effects [25,26]. Any of these factors might influence the shape of the dose-response function.

Moreover, the progression from cellular damage to whole organism diseases that affect mortality, like cancer, involves complex processes that are not completely understood [27,28]. Even if the most relevant forms of cellular level damage have linear-quadratic or linear-quadratic-exponential dose responses, it is not clear that the long-term dose-responses would take the same form.

At present, there is enough evidence to reject the linear-quadratic model used by BEIR VII and other reports, but not enough evidence to confidently extrapolate the true shape of long term organism-level dose-responses based on existing knowledge of cellular and tissue level responses.

### Estimating the risk of low dose and protracted exposures with minimal assumptions

How can protection agencies estimate the relative risk of low dose and protracted exposures using atomic bomb survivor data given the existing uncertainty about the relationship between radiation exposure and long-term health responses? Rather than attempting to derive the most plausible dose-response model and base all estimates upon it, we believe that low dose and protracted risk estimates should be based on a model with minimal assumptions in order to minimize the odds of making wrong assumptions.

With this in mind, we propose that future estimates of the relative risk of protracted exposures, made up of many low dose exposures that sum to a moderate total dose, should be simple and direct. The relative risk of such exposures should not be based on the apparent shape of acute dose-responses. Instead, it should be based on direct comparisons of acute vs. protracted responses. Linear fits to acute exposures should be compared to linear fits to protracted exposures to estimate a difference in slope. Linear fits to the data are the best to use, not because the true dose response is necessarily linear, but because the shape of the true dose response is unknown, while linear dose-responses fit observed data quite well, and because these factors are applied to linear fits of atomic bomb survivor data. It is also important that estimates of a protraction factor specifically account for differences in age at exposure, which is typically higher, on average, in animals that are exposed to protracted radiation than the acutely exposed animals they are compared to.

Estimating the relative risk of these low dose exposures, on the other hand, continues to be challenging. Ideally, it would be based on direct comparisons between acute high dose exposures and acute low dose exposures. Unfortunately, the statistical power of such analyses is low because the effect of such low dose exposures is small. Therefore, the estimated risk of low dose exposures will continue to depend on the assumption of some theoretical dose response function. Most radiation protection policy is based on the argument that the risk following such low dose exposures is collinear the risk following protracted exposures. This is because protracted exposures are composed of many small low dose exposures separated in time. However, one could also plausibly argue that the risk following low dose exposures should be collinear with the acute dose response or something else all together. We will not attempt to make a specific recommendation in this paper, except to note that if low dose and protracted dose responses are collinear, then improving the estimate of the effects of fractionated exposures (which remain uncertain) will improve the estimate of the effects low dose exposures as well.

Finally, future attempts to derive a protraction factor should be based on larger datasets. Larger datasets, like the one used in this report, can either improve the precision of an estimate or expose flaws in the method used to estimate it. Either would improve the state of radiation protection.

We recommend four ways to increase the size of the dataset used to estimate the risk of protracted exposures.

One possible approach is to include data from radiation exposures greater than 1.5 Sv, the maximum dose allowed in the BEIR VII analysis of animal mortality data. Specifically, data should be considered across the dose range from 0–3 Sv, the ranges of data that have historically been considered when estimating acute exposure risk from atomic bomb survivor data [8,51,52]. This is an important step because it aligns protraction factor estimates to the atomic bomb survivor dose response estimates to which these factors are applied.

A second approach is to analyze carcinogenesis data in addition to life shortening data. Our analysis was focused on life shortening radiation effects because lifespan is a critical health outcome that is consistently recorded across many studies. However, radiation protection efforts focus primarily on increases in carcinogenesis, because this is the primary long-term health effect of radiation exposure. It would be valuable to estimate protraction effects based on carcinogenesis, and especially different types of cancer, which may respond differently to protraction.

A third way is to combine results of human observations with animal studies. Our analysis has focused on animal data because these are the data accessible to us. We encourage other groups with access to human data to estimate protraction factors using a combination of human and animal data. Especially important is the question of whether the effect of protraction is significantly different in human data when compared to animal data. Jacob’s observations suggest that this may be true [11], though uncertainty remains high. Without a unified analysis it is difficult to quantify how plausible this possibility is.

A final way to increase the amount of animal data available for analysis is to increase the number of animal studies included in publicly available archives. There have been hundreds of large studies on the lifelong effects radiation exposure conducted on a variety of animal species. Many of these studies have been included in publicly accessible archives like the ERA and the Janus archives. Nevertheless, much data are still inaccessible to the public. We encourage the research community to make their radiation animal data publicly available in order to enable the development of better radiation protection standards.

## Conclusion

We find that the linear-quadratic dose response model used by BEIR VII to estimate DDREF_LSS_ is inconsistent with the observed data. Particularly, curvatures in dose response following acute exposures do not suggest a DDREF_LSS_ correction, while comparisons of acute and protracted exposures do.

We find evidence that protracted exposures are safer than current estimates suggest. A range of models suggests that protracted exposures are between 2.0 and infinitely times safer with 95% confidence (Table 2). This result supports Hoel's analysis suggesting that BEIR VII's DDREF_LSS_ estimate is too low [18]. It also contradicts Jacob et al. findings that show that protracted exposures to radiation workers were as dangerous as acute exposures to atomic bomb survivors [11]. However, these results are based on the small number of studies that directly compared acute and protracted exposures and also met the BEIR VII inclusion criteria. The observed attenuation of risk may be due to the average age at which animals were given protracted exposures and may not be a direct effect of protraction.

We propose that future estimates of the risk of low dose and protracted exposures should be based on additional studies that directly compare acute and protracted exposures over a wider range of exposures, accounting for the effects of age at exposure, including both human and animal observations, and including doses across the range of doses analyzed from atomic bomb survivor data, 0 to 3 Sv. We encourage the use of carcinogenesis data in addition to the life-shortening data we present here. Because of uncertainty in the factors that influence long term health effects following radiation exposure, we encourage continued debate over the question of whether the risk following low-dose exposures is collinear with the risk following protracted exposures. Finally, we encourage radiation researchers to contribute more animal studies to existing publicly accessible radiobiology archives to improve future efforts to model radiation risk.

## Supporting Information

S1 Table Data that could not be confirmed in the literature. Several treatment groups were excluded from the analysis because the ERA or Janus data could not be confirmed in primary literature. The reason(s) for exclusion is listed for each treatment group. ERA study group identifiers denote treatment groups.
(DOCX)

S2 Table Data concordance. A description of data used in this analysis. The first column details the data sources stratified by sex, strain, quality of radiation, and age at first exposure. Strata are organized from most animals (top) to least (bottom) and numbered 1–16 corresponding with the figures in this paper. Also listed are the ERA study IDs corresponding to the data in the strata and references to these studies in the literature. Subsequent columns are further grouped by treatment so that they share the same total dose (Gy), dose-rate (Gy/min), distinct fractions (fr.), and interval between fractions in days (int.). These groups correspond to individual data-points and lines used in the figures in this paper. Total number of animals (n), average lifespan (μ age), and the standard error of the mean lifespan (σ) are shown for each treatment group. Note: In some analysis data were also stratified by study, which excluded several control groups in strata 2 and 3 that came from different studies at ANL.
(DOCX)

S3 Table DDREF_LSS_ estimates by strata. Estimates of α, β, and the corresponding DDREFLSS (with 95% credible intervals) for each stratum analyzed independently using the BEIR VII linear-quadratic model.
(DOCX)
