# Supplementary material for: The Increase in Animal Mortality Risk following Exposure to Sparsely Ionizing Radiation Is Not Linear Quadratic with Dose
Source: PLoS One. 2015 Dec 9;10(12):e0140989. doi: 10.1371/journal.pone.0140989 (PMC4674094; doi:10.1371/journal.pone.0140989)
Supplement: S1 Table — Several treatment groups were excluded from the analysis because the ERA or Janus data could not be confirmed in primary literature. The reason(s) for exclusion is listed for each treatment group. ERA study group identifiers denote treatment groups. (DOCX) [file pone.0140989.s001.docx]

1. ­

| **Treatment group**   1. **ERA identifier** | 1. **Reason for exclusion** |
| --- | --- |
| 1. 1003-21-6 | 1. This treatment group was abandoned. Cause of death is listed as 'abandon' or 'remove to another experiment'. |
| 1. 11-2-79 2. 11-2-80 3. 11-2-81 | 1. Some mice in these treatment groups had impossibly long lifespans, e.g. 6993. This seems to be a coding error in the data. No access to the correct data was available. |
| 1. 1007-3-8 2. 1007-3-16 | 1. Mean lifespans differed from those reported in Table 1 of [1] by more than 1 standard deviation. Moreover, there are fewer mice in the ERA dataset than listed by Ullrich and Storer. |
| 1. 3-4  (all treatments) | 1. These groups are identical to those listed in study 3-2. |
| 1. 11-1 2. (all treatments) | 1. No external data source was found to confirm the treatments and lifespans in this study. |
| 1. 11-2 2. (all treatments) | 1. No external data source was found to confirm the treatments and lifespans in this study. |
| 1. 3-2 2. (all treatments) | 1. No external data source was found to confirm the treatments and lifespans in this study. The only source found that details this study [2] was limited to neutron exposures. |
| 1. 1003-xx | 1. No external data source was found to confirm the treatments and lifespans in this study. |
| 1. 9-8 | 1. No external data source was found to confirm the treatments and lifespans in this study. |

1. **S1 Table: Data that could not be confirmed in the literature**
   Several treatment groups were excluded from the analysis because the ERA or Janus data could not be confirmed in primary literature. The reason(s) for exclusion is listed for each treatment group. ERA study group identifiers denote treatment groups.

**References**

1. Ullrich RL, Storer JB. Influence of gamma irradiation on the development of neoplastic disease in mice. III. Dose-rate effects. Radiat Res. 1979;80: 325–342. doi:10.2307/3575061

2. Di Majo V, Coppola M, Rebessi S, Saran a, Pazzaglia S, Pariset L, et al. Neutron-induced tumors in BC3F1 mice: effects of dose fractionation. Radiat Res. 1994;138: 252–259. doi:10.2307/3578595
