# Supplementary material for: The Increase in Animal Mortality Risk following Exposure to Sparsely Ionizing Radiation Is Not Linear Quadratic with Dose
Source: PLoS One. 2015 Dec 9;10(12):e0140989. doi: 10.1371/journal.pone.0140989 (PMC4674094; doi:10.1371/journal.pone.0140989)
Supplement: S2 Table — A description of data used in this analysis. The first column details the data sources stratified by sex, strain, quality of radiation, and age at first exposure. Strata are organized from most animals (top) to least (bottom) and numbered 1–16 corresponding with the figures in this paper. Also listed are the ERA study IDs corresponding to the data in the strata and references to these studies in the literature. Subsequent columns are further grouped by treatment so that they share the same total dose (Gy), dose-rate (Gy/min), distinct fractions (fr.), and interval between fractions in days (int.). These groups correspond to individual data-points and lines used in the figures in this paper. Total number of animals (n), average lifespan (μ age), and the standard error of the mean lifespan (σ) are shown for each treatment group. Note: In some analysis data were also stratified by study, which excluded several control groups in strata 2 and 3 that came from different studies at ANL. (DOCX) [file pone.0140989.s002.docx]

| **Strata in this analysis**  Stratum id, sex, strain, quality, age at first exposure, ERA study ids, and references | **n** | **age**  **(μ +/- σ)** | **Gy** | **Gy/min** | **fr.** | **int.** |
| --- | --- | --- | --- | --- | --- | --- |
| 1 - ♀ RFM/Un Mice ORNL γ-ray at 70 days old  1007-3  [1] | 2696 | 632 +/- 3.2 | 0.1 | 0.45 | 1 | - |
|  | 930 | 614 +/- 5.3 | 0.25 | 0.45 | 1 | - |
|  | 1064 | 553 +/- 5.3 | 0.5 | 0.45 | 1 | - |
|  | 237 | 541 +/- 11.2 | 0.75 | 0.45 | 1 | - |
|  | 1045 | 538 +/- 5.4 | 1 | 0.45 | 1 | - |
|  | 1005 | 487 +/- 5.8 | 1.5 | 0.45 | 1 | - |
| 2 - ♀ B6CF1 Mice ANL γ-ray at 114 days old  1003-20, 21, 22, 24, 25, 26, and 30  [2] | 3852 | 969 +/- 3.1 | 0 | 0 | 1 | - |
|  | 497 | 963 +/- 8.7 | 0.22 | 0.011 | 1 | - |
|  | 346 | 968 +/- 10.8 | 0.43 | 0.022 | 1 | - |
|  | 791 | 919 +/- 6.6 | 0.86 | 0.043 | 1 | - |
|  | 598 | 957 +/- 7.7 | 1 | 0.001 | 60 | 7 |
| 3 - ♂ B6CF1 Mice ANL γ-ray at 113 days old  1003-20, 21, 22, 24, 26, 28, 29, and 30  [2] | 3275 | 986 +/- 3.4 | 0 | 0 | 1 | - |
|  | 585 | 938 +/- 8.1 | 0.86 | 0.043 | 1 | - |
|  | 594 | 971 +/- 7.5 | 1 | 0.001 | 60 | 7 |
|  | 160 | 939 +/- 16.4 | 1.37 | 0.069 | 1 | - |
| 4 - ♂ C57BL/Cnb Mice SCK/CEN γ-ray at 84 days old  9-6  [3,4] | 467 | 613 +/- 7.1 | 0 | 0 | 1 | - |
|  | 241 | 581 +/- 9.5 | 0.25 | 0.3 | 1 | - |
|  | 107 | 605 +/- 15.6 | 0.25 | 0.3 | 10 | 1 |
|  | 236 | 564 +/- 10.2 | 0.5 | 0.3 | 1 | - |
|  | 109 | 604 +/- 14.3 | 0.5 | 0.3 | 10 | 1 |
|  | 241 | 550 +/- 9.6 | 1 | 0.3 | 1 | - |
|  | 104 | 622 +/- 16.7 | 1 | 0.3 | 8 | 0.13 |
|  | 115 | 615 +/- 13.6 | 1 | 0.3 | 10 | 1 |
| 5 - ♂ RFM/Un Mice ORNL γ-ray at 70 days old  1007-3  [1] | 430 | 711 +/- 7.7 | 0 | 0 | 1 | - |
|  | 256 | 720 +/- 10.9 | 0.1 | 0.45 | 1 | - |
|  | 94 | 711 +/- 18.1 | 0.25 | 0.45 | 1 | - |
|  | 247 | 680 +/- 11.4 | 0.5 | 0.45 | 1 | - |
|  | 230 | 673 +/- 11.9 | 1 | 0.45 | 1 | - |
|  | 199 | 651 +/- 13.8 | 1.5 | 0.45 | 1 | - |
| 6 - ♂ BALB/c/Cnb Mice SCK/CEN γ-ray at 84 days old  9-5  [3–5] | 322 | 766 +/- 8.9 | 0 | 0 | 1 | - |
|  | 191 | 745 +/- 13.5 | 0.25 | 4 | 1 | - |
|  | 111 | 778 +/- 12.8 | 0.25 | 4 | 10 | 1 |
|  | 194 | 736 +/- 13.7 | 0.5 | 4 | 1 | - |
|  | 110 | 740 +/- 16.2 | 0.5 | 4 | 10 | 1 |
|  | 191 | 732 +/- 10.7 | 1 | 4 | 1 | - |
|  | 113 | 751 +/- 15.9 | 1 | 4 | 10 | 1 |
| 7 - ♀ BC3F1 Mice ENEA X-ray at 91 days old  3-1  [6,7] | 632 | 878 +/- 6 | 0 | 0 | 1 | - |
|  | 100 | 912 +/- 17 | 0.04 | 0.06 | 1 | - |
|  | 84 | 893 +/- 18.3 | 0.08 | 0.06 | 1 | - |
|  | 53 | 854 +/- 21.8 | 0.16 | 0.06 | 1 | - |
|  | 58 | 874 +/- 24.6 | 0.32 | 0.06 | 1 | - |
|  | 57 | 833 +/- 21 | 0.64 | 0.64 | 1 | - |
|  | 60 | 707 +/- 23 | 1.28 | 0.64 | 1 | - |
| 8 - ♂ C57BL/6Bd Mice ORNL γ-ray at 70 days old  1007-2  [1] | 502 | 906 +/- 6.1 | 0 | 0 | 1 | - |
|  | 254 | 909 +/- 8.5 | 0.5 | 0.4 | 1 | - |
|  | 260 | 922 +/- 8.2 | 1 | 0.4 | 1 | - |
| 9 - ♀ C3Hf/Bd Mice ORNL γ-ray at 70 days old  1007-2  [1] | 501 | 778 +/- 5.8 | 0 | 0 | 1 | - |
|  | 249 | 727 +/- 6.9 | 0.5 | 0.4 | 1 | - |
|  | 250 | 693 +/- 7.6 | 1 | 0.4 | 1 | - |
| 10 - ♀ C57BL/6Bd Mice ORNL γ-ray at 70 days old  1007-2  [1] | 491 | 858 +/- 6.9 | 0 | 0 | 1 | - |
|  | 253 | 855 +/- 11.2 | 0.5 | 0.4 | 1 | - |
|  | 251 | 865 +/- 10.3 | 1 | 0.4 | 1 | - |
| 11 - ♂ C3Hf/Bd Mice ORNL γ-ray at 70 days old  1007-2  [1] | 502 | 732 +/- 5.8 | 0 | 0 | 1 | - |
|  | 244 | 713 +/- 7.9 | 0.5 | 0.4 | 1 | - |
|  | 248 | 721 +/- 8.7 | 1 | 0.4 | 1 | - |
| 12 - ♂ BC3F1 Mice ENEA X-ray at 92 days old  3-5  [6,7] | 430 | 824 +/- 8.8 | 0 | 0 | 1 | - |
|  | 44 | 828 +/- 27.4 | 0.5 | 0.133 | 1 | - |
|  | 48 | 797 +/- 34.4 | 1 | 0.133 | 1 | - |
| 13 - ♂ C57BL/Cnb Mice SCK/CEN X-ray at 7 days old  9-7  [3–5] | 105 | 757 +/- 13.7 | 0 | 0 | 1 | - |
|  | 72 | 777 +/- 21.4 | 0.5 | 1 | 1 | - |
|  | 70 | 810 +/- 16.1 | 1 | 1 | 1 | - |
| 14 - ♂ BC3F1 Mice ENEA X-ray at -4 days old  3-5  [6,7] | 34 | 853 +/- 42.3 | 0 | 0 | 1 | - |
|  | 48 | 799 +/- 26.1 | 0.3 | 0.133 | 1 | - |
|  | 61 | 822 +/- 27.4 | 0.9 | 0.133 | 1 | - |
|  | 46 | 897 +/- 25.4 | 1.5 | 0.133 | 1 | - |
| 15 - ♀ BC3F1 Mice ENEA X-ray at -4 days old  3-5  [6,7] | 39 | 866 +/- 25.7 | 0 | 0 | 1 | - |
|  | 40 | 883 +/- 37.2 | 0.3 | 0.133 | 1 | - |
|  | 44 | 850 +/- 22.2 | 0.9 | 0.133 | 1 | - |
|  | 50 | 872 +/- 30.6 | 1.5 | 0.133 | 1 | - |
| 16 - ♂ BC3F1 Mice ENEA X-ray at 580 days old  3-5  [6,7] | 41 | 886 +/- 21.9 | 0 | 0 | 1 | - |
|  | 42 | 901 +/- 21.8 | 0.5 | 0.133 | 1 | - |
|  | 43 | 874 +/- 20.2 | 1 | 0.133 | 1 | - |

**S2 Table: Data concordance**

A description of data used in this analysis. The first column details the data sources stratified by sex, strain, quality of radiation, and age at first exposure. Strata are organized from most animals (top) to least (bottom) and numbered 1-16 corresponding with the figures in this paper. Also listed are the ERA study IDs corresponding to the data in the strata and references to these studies in the literature. Subsequent columns are further grouped by treatment so that they share the same total dose (Gy), dose-rate (Gy/min), distinct fractions (fr.), and interval between fractions in days (int.). These groups correspond to individual data-points and lines used in the figures in this paper. Total number of animals (n), average lifespan (μ age), and the standard error of the mean lifespan (σ) are shown for each treatment group. Note: In some analysis data were also stratified by study, which excluded several control groups in strata 2 and 3 that came from different studies at ANL.

**References**

1. Ullrich RL, Storer JB. Influence of gamma irradiation on the development of neoplastic disease in mice. III. Dose-rate effects. Radiat Res. 1979;80: 325–342. doi:10.2307/3575061

2. Grahn D, Wright B, Carnes B, Williamson S, Fox C. Studies of acute and chronic radiation injury at the biological and medical research division, Argonne National Laboratory, 1970-1992: The JANUS program survival and pathology data [Internet]. Argonne IL; 1995. Available: https://s3.amazonaws.com/janus-cloud2/www/janus2/reports/complete.pdf

3. Maisin JR, Wambersie A, Gerber GB, Mattelin G, Lambiet-Collier M, Gueulette J. The effects of fractionated gamma irradiation on life shortening and disease incidence in BALB/c mice. Radiat Res. 1983;94: 359–373. doi:10.2307/3575970

4. Maisin JR, Gerber GB, Vankerkom J, Wambersie A. Survival and diseases in C57BL mice exposed to X-rays or at an age of 7 or 21 days. Radiat Res. 1996;146: 453–460. doi:10.2307/3579307

5. Maisin JR, Wambersie A, Gerber GB, Mattelin G, Lambiet-Collier M, DeCoster B, et al. Life-shortening and disease incidence in C57Bl mice after single and fractionated γ and high energy neutron exposure. Radiat Res. 1988;113: 300–317. doi:10.2307/3577205

6. Covelli V, Majo V Di, Bassani B, Rebessi S, Coppola M, Silini G. Influence of age on life shortening and tumor induction after X-ray and neutron irradiation. Radiat Res Soc. 1984;100: 348–364. doi:10.2307/3576356

7. Covelli V, Coppola M, Majo V Di, Rebessi S, Bassani B. Tumor induction and life shortening in BC3F1 female mice at low doses of fast neutron and X-rays. Radiat Res. 1988;113: 362–374. doi:10.2307/3577210
