# Supplementary material for: The Increase in Animal Mortality Risk following Exposure to Sparsely Ionizing Radiation Is Not Linear Quadratic with Dose
Source: PLoS One. 2015 Dec 9;10(12):e0140989. doi: 10.1371/journal.pone.0140989 (PMC4674094; doi:10.1371/journal.pone.0140989)
Supplement: S3 Table — Estimates of α, β, and the corresponding DDREFLSS (with 95% credible intervals) for each stratum analyzed independently using the BEIR VII linear-quadratic model. (DOCX) [file pone.0140989.s003.docx]

# ­­

| **Stratum** | **DDREF_LSS_** | **α (×10^-5^)** | **β (×10^-5^)** |
| --- | --- | --- | --- |
| 1. ♀ RFM/Un mice ORNL γ-ray at 70 days old | 0.9 (0.7, 1.5) | 56 | -5.6 |
| 2. ♀ B6CF1 mice ANL γ-ray at 114 days old | ∞ (0.7, ∞) | 0.0 | 7.4 |
| 3. ♂ B6CF1 mice ANL γ-ray at 113 days old | 3.3 (0.7, ∞) | 2.1 | 4.8 |
| 4. ♂ C57BL/Cnb mice SCK/CEN γ-ray at 84 days old | ∞ (0, ∞) | 0.0 | 22 |
| 5. ♂ RFM/Un mice ORNL γ-ray at 70 days old | 0.8 (0.5, ∞) | 23 | -4.7 |
| 6. ♂ BALB/c/Cnb mice SCK/CEN γ-ray at 84 days old | 0.6 (0, ∞) | 5.9 | -2.3 |
| 7. ♀ BC3F1 mice ENEA X-ray at 91 days old | ∞ (1.6, ∞) | 0.0 | 21 |
| 8. ♂ C57BL/6Bd mice ORNL γ-ray at 70 days old | ∞ (0, ∞) | 0.0 | -2.3 |
| 9. ♀ C3Hf/Bd mice ORNL γ-ray at 70 days old | 2.2 (0.4, ∞) | 7.5 | 9.3 |
| 10. ♀ C57BL/6Bd mice ORNL γ-ray at 70 days old | ∞ (0, ∞) | 0.0 | 2.1 |
| 11. ♂ C3Hf/Bd mice ORNL γ-ray at 70 days old | ∞ (0, ∞) | 0.0 | 3.8 |
| 12. ♂ BC3F1 mice ENEA X-ray at 92 days old | ∞ (1, ∞) | 0.0 | 12.8 |
| 13. ♂ C57BL/Cnb mice SCK/CEN X-ray at 7 days old | ∞ (0.5, ∞) | 0.0 | -18 |
| 14. ♂ BC3F1 mice ENEA X-ray at -4 days old | 2.7 (0.6, ∞) | -4.7 | -8.1 |
| 15. ♀ BC3F1 mice ENEA X-ray at -4 days old | 0.3 (0, 0.6) | -4.3 | 3.2 |
| 16. ♂ BC3F1 mice ENEA X-ray at 580 days old | ∞ (0, ∞) | 0.0 | 1.8 |

**S3 Table: DDREF_LSS_ estimates by strata**

Estimates of α, β, and the corresponding DDREF_LSS_ (with 95% credible intervals) for each stratum analyzed independently using the BEIR VII linear-quadratic model
